# Neurological teaching in times of crisis

**DOI:** 10.3205/zma001362

**Published:** 2020-12-03

**Authors:** Anne-Sophie Biesalski, Isabelle von Kirchbauer, Friederike Schmidt-Graf

**Affiliations:** 1 Ruhr-Universität Bochum, Neurologische Klinik St. Josef Hospital Bochum, Bochum, Germany; 2 Technische Universität München, Klinikum rechts der Isar, TUM Medical Education Center, Munich, Germany; 3 Technische Universität München, Klinikum rechts der Isar, Klinik und Poliklinik für Neurologie, Munich, Germany

**Keywords:** medical education, medical curriculum, neurology, corona, teaching methods, e-learning

## Abstract

**Background/Objective: **The changes to medical studies that became necessary as part of the Corona pandemic have also forced considerable adjustments in Neurology. Classroom teaching had to be converted almost entirely to digital formats within a short period of time. The present study provides an overview of the respective changes and associated complications as well as opportunities in teaching Neurology.

**Methodology: **Lecturers in Neurology at all University hospitals in Germany were asked about their approach and the individual changes in their curriculum. Of a total of 39 locations, 23 answered the online questionnaire (see attachment 1).

**Results: **While frontal teaching and lectures could be carried out digitally without any problems, difficulties arose especially in learning physical examination and bedside teaching. Most of the participants stated that they had not found satisfactory e-learning formats to replace teaching at the patients bed.

**Conclusion: **The ad hoc changes in teaching Neurology resulted in significant additional effort for the part of lecturers, but were generally well accepted by students. The Corona pandemic thus ultimately offers an opportunity to enrich teaching in Neurology.

## Zusammenfassung

**Hintergrund/Zielsetzung:** Die im Rahmen der Corona-Pandemie notwendig gewordenen Veränderungen des Medizinstudiums haben auch in der Neurologie erhebliche Anpassungen erzwungen. Die Präsenzlehre musste innerhalb kurzer Zeit nahezu vollständig auf digitale Formate umgestellt werden. Die vorliegende Studie gibt einen Überblick über die jeweiligen Veränderungen und hiermit verbundenen Komplikationen wie auch Chancen in der neurologischen Lehre.

**Methodik: **Die Lehrbeauftragten Neurolog*Innen aller Universitätskliniken in der Bundesrepublik Deutschland wurden zu Ihrem Umgang und den individuellen Neuerungen im Lehrplan befragt. Von insgesamt 39 Standorten beantworteten 23 den Online-Fragebogen (siehe Anhang 1).

**Ergebnisse: **Während Frontalunterricht und Vorlesungen insgesamt problemlos digital durchgeführt werden konnten, ergaben sich insbesondere im Untersuchungskurs sowie dem Unterricht am Patientenbett Schwierigkeiten. Die Befragten gaben überwiegend an, keine zufriedenstellenden E-Learning-Formate als Ersatz für das Bedside-Teaching gefunden zu haben.

**Schlussfolgerung: **Die ad Hoc-Umstellung der neurologischen Lehre hatte zumeist einen deutlichen Mehraufwand auf Seiten der Lehrenden zur Folge, wurde von den Studierenden jedoch insgesamt gut angenommen. Die Corona-Pandemie bietet damit letztlich eine Chance, die Lehre in der Neurologie zu bereichern.

## Background

The Corona pandemic did also lead to dramatic changes in medical teaching. Since at least when the contact restrictions came into effect in March 2020, medical faculties throughout Germany also decided to temporarily suspend classroom teaching. For that, digital formats gained importance and quickly found their way into medical education.

## Methodology

At the end of summer semester 2020, the teaching staff of neurologists at all University hospitals were asked about the feasibility and benefits of new e-learning formats during the Corona crisis using an online questionnaire (see attachment 1 ).

The questionnaire (see attachment 1 ) contained 21 items and a Likert scale of 6 parts. The subsequent evaluation was done using Evasys. The free text fields were evaluated qualitatively and categorially.

## Results

By the end of a 2 weeks survey period, 23 neurologists from 39 University hospitals had participated.

For more than one third of the respondents (71.4%), the beginning of the semester had to be postponed. Only 28.6% stated that the semester had started at the planned date. Teaching rounds (60.9%), practical courses (52.2%), internships/hospitations (39.1%) as well as seminars and small group lessons (43.5%) were cancelled without replacement. Lectures could be completely replaced digitally (100%). For this purpose, mainly slides (78.3%) or lecture recordings (65.2%) were created, lectures were given via Zoom (69.6%), and online platforms of the Universities (43.5%) and Moodle courses (47.8%) were used. The attendance at compulsory digital courses was checked in about half of the cases (47.8% vs. 52.2% without checking attendance). Failed exams were not reported; the majority (78.3%) took place in attendance and in compliance with hygiene regulations.

Almost half (47.8%) of the respondents stated that they had sufficient or particularly good (21.7%) digital knowledge and skills prior to Corona, while 30.4% had them only to a small extent. E-learning formats existed already at 56.6% of the sites.

The time expenditure for teaching was increased in the summer semester 2020: 81.8% stated that they had needed more or significantly more time and effort than usual.

The Likert scales showed that ad hoc digitalization was not perceived without any problems overall, but it was seen as some progress for teaching. The lecturers themselves have benefited from digitalization, although in their opinion the students have not had any extraordinarily positive benefit and the interest in Neurology has apparently not increased. For the next semester, the respondents would like to see more face-to-face teaching even under the given conditions: especially practical courses and bedside teaching, more personnel and professional/technical support, and improvements in digital formats.

The free text informations on special courses during the Corona period showed a certain wealth of ideas: the offers ranged from a internship with a safety distance of 1.5 m between all participants to practical courses with examination of the lecturers among themselves as well as an elective Covid-19 module.

## Conclusion

In Neurology, a high degree of flexibility and rapid implementation in adaptation of teaching formats is evident.

At most Universities, e-learning formats already existed in addition to classroom teaching, which could easily be expanded. Nevertheless, the changes in teaching process meant considerable additional work for the physicians in charge of teaching - for example in creating digital lectures or administration of online materials. Especially, the digitalization of the neurological examination course was experienced as a challenge.

In personal conversations with teachers and lecturers, the overall view was positive: many reported higher attendance at elective sessions and more targeted inquiries in chats or via e-mail, which ultimately led to a more individualized discussion. This is also shown by surveys of the University of Lübeck [1], as well as studies on training centered on learners [2], [3]. The availability and uncomplicated usability of teaching materials, regardless of location, also argue for the digitalization of neurological teaching and meet with the approval of the generation of students who are interested in technology, anyway. The positive effects of multimedia teaching have already been demonstrated many times in Neurology [4] and digitalization offers considerable potential for medical studies, possibly fundamentally changing medical education as a whole [5].

Ultimately, Corona has led to far-reaching changes in teaching at all locations. Would it be unreasonable to say that the pandemic has done us some service? The digitalization that was ultimately forced upon us has made possible to take a step towards modern teaching that previously seemed impossible in many places due to rigid structures, funding agencies, administrative apparatus and a "whatever it takes" mentality.

Especially in clinical Neurology, teaching at the patient's bedside will not be possible to do without. However, in the future considerably more digital formats can be used. Hybrid events with blended learning units are conceivable, e.g. in practical courses: after a digital teaching and learning phase at home or in peer-teaching, the examination can take place more effectively and with better prepared students at the patient's bed. Such curricular changes would tie up fewer personnel resources in the long term and help to convey a uniform standard. In this respect, the Corona pandemic paves the way for flexible, future-oriented formats of neurological teaching and can enrich teaching and stimulate further innovation.

## Competing interests

The authors declare that they have no competing interests. 

## Supplementary Material

Questionnaire: Teaching in times of Corona
