# Supplementary material for: Neurological teaching in times of crisis
Source: GMS J Med Educ. 2020 Dec 3;37(7):Doc69. doi: 10.3205/zma001362 (PMC7740022; doi:10.3205/zma001362)
Supplement: Questionnaire: Teaching in times of Corona [file JME-37-69-s-001.pdf]

Attachment 1: Questionnaire: Teaching in times of Corona

Please indicate which lessons usually take place in clinical Neurology at your location (multiple answers possible):

- Lecture
- Seminar / small group lessons
- Physical examination course/ Bedside course
- Teaching rounds
- Work shadowing / Hospitations
- Clinical Internship
- Other: Free text

Have you already had digital or e-learning formats before Corona?

- Yes, which ones? Free text
- No

Have you ever gained e-learning experience at your location yourself? What experiences have you had with it?

Free text

Which lessons could / could not take place at your location due to Corona restrictions and were NOT replaced (multiple answers possible)?

- Lectures
- Seminar / small group lessons
- Physical examination course/ Bedside course
- Teaching rounds
- Work shadowing / Hospitations
- Clinical Internship
- Other: Free text

Which lessons can/could be digitally implemented at your location (multiple answers possible)?

- Lectures

- Seminar / small group lessons

- Physical examination course/ Bedside course

- Teaching rounds

- Work shadowing / Hospitations

- Clinical Internship

- Other: Free text

In which year do Neurology lessons normally take place at your location (multiple answers possible)?

- Pre-clinical semester (1st - 4th semester)

- 1st clinical year of study

- 2nd clinical year of study

- 3rd clinical year of study

- Final year

Was it possible to start the past semester at the scheduled time?

- Yes

- No

Has the participation of students in the now digitally guided compulsory courses been checked?

- Yes

- No

- Other procedure: Free text

How do you currently rate the learning effect of students in Neurology (practical impementation excluded)?

- The students learn significantly less than in semesters with attendance
- Students learn less than semesters with attendance
- Students learn just as much - just differently
- Students benefit from the changed situation - they learn more than usual

The certificates in Neurology are issued with (multiple answers possible):

- Digital presence
- Without proof
- Exam
- Other: Free text

How are exams in Neurology currently performed at your location?

- The exams are currently suspended / will take place in the following semester
- The exams are conducted digitally (e.g. via video conferencing, online exam)
- The tests are carried out on site, in compliance with the specified safety restrictions
- The exams are replaced (e.g. by seminar papers / other tasks)

How do you rate your digital knowledge and skills before the Corona crisis?

- Not available
- Very low (e.g. limited creation of a PowerPoint presentation, uploading of files to a learning management software not possible)
- Low (e.g. moderate skills in using Microsoft Office, uploading of files possible, no in-depth knowledge e.g. in creating/using videos, no experience with video conferencing or similar)
- Adequate (e.g. all common digital formats (MS Office, Moodle, Video etc.) are mastered, help is sometimes required)
- Very good (easy handling and regular use of all common digital formats, uncomplicated learning of new content)

How do you currently rate your digital knowledge and skills?

- Not available
- Very low (e.g. limited creation of a PowerPoint presentation, uploading of files to a learning management software not possible)
- Low (e.g. moderate skills in using Microsoft Office, uploading of files possible, no in-depth knowledge e.g. in creating/using videos, no experience with video conferencing or similar)
- Adequate (e.g. all common digital formats (MS Office, Moodle, Video etc.) are mastered, help is sometimes required)
- Very good (easy handling and regular use of all common digital formats, uncomplicated learning of new content)

What software and techniques were used for digital teaching at your location (multiple answers possible)?

- Zoom
- Skype
- Google
- Adobe Connect
- Moodle
- Own platforms of the University
- Screencast
- Laying technique
- Stop Motion
- Recording lectures
- Uploading slides
- Other: Free text

What helpful support did you receive when implementing new teaching formats?

- Didactics of the medical faculty (Institute/ Department)

- Didactics department of the University

- computer scientist

- Student assistants, e-scouts... or similar

- Environment: friends/ colleagues

- Self-study on the Internet

- No support, no training

Are there currently opportunities for practical teaching at your location?

- Teaching or rounds for final year students

- Work shadowing

- Internship/ bedside course

- Others

- Currently no practical lessons

How do you rate the time spent on teaching and preparing lessons in the past semester?

- Less effort than before

- About the same effort than before

- More effort than before

- Significantly more effort than before

Please, evaluate the following statements:

Rating with 5-1: 5fully agree - agree - neutral - agree less - disagree1

- The Corona-related changes mean overall progress for teaching at my location

- The teaching in Neurology at my location could be digitalized without any problems

- The "Corona semesters" are lost semesters in Neurology - students are not sufficiently prepared for the demands of clinical Neurology

- I learned a lot through the need to digitize teaching and “benefited” from the changes

- The Corona-related changes in teaching have a positive effect on students' knowledge

- The interest of students in Neurology is greater than in previous semesters thanks to the digital offerings

- The students give positive feedback on the current changes in neurological teaching at my location

What would you like to see/change for the next (at least semi-digital) semester?

Free text

Other comments?

Free text

What level of education do you have?

- Senior physician

- Specialist

- Assistant doctor

- Others

How old are you?

- 25-35 years

- 35-45 years

- 45-55 years

- 55-65 years

- >65 years
